# BARRIERS AND FACILITATORS TO IMPLEMENTING COMMUNITY FIRST CHOICE

**DOI:** 10.1093/geroni/igz038.2837

**Published:** 2019-11-08

**Authors:** Lisa Beauregard, Edward A Miller

**Affiliations:** University of Massachusetts Boston, Boston, Massachusetts, United States

## Abstract

Community First Choice is a program within the Affordable Care Act that encourages states to expand Medicaid home and community-based services (HCBS). Specifically, this Medicaid state plan benefit provides states with an additional 6% federal match to promote greater rebalancing of long-term services and supports. Through Community First Choice, states can offer services that assist with activities of daily living, instrumental activities of daily living, and health-related tasks. The program is optional for states, and, to date, eight states have pursued Community First Choice. The purpose of this study is to understand the barriers and facilitators to implementing Community First Choice in two states. Data was collected through semi-structured interviews with individuals involved in HCBS policy nationally and in Maryland and Texas, including government bureaucrats, consumer advocates, and provider representatives. The results suggest that communication with the Centers for Medicare and Medicaid Services, the enhanced federal match, and leveraging existing HCBS infrastructure facilitated implementation. Maryland and Texas encountered challenges implementing Community First Choice because of constraints posed by existing HCBS programs, ambitious timelines, limited staff resources, and insufficient engagement with external stakeholders. The findings suggest that implementing Community First Choice is a large undertaking, and states should ensure they have enough time and sufficient staffing for the implementation process. States should also understand how implementing Community First Choice will impact existing HCBS offerings and how leveraging HCBS infrastructure can facilitate implementation. The lessons from implementing Community First Choice can be informative to other states pursuing or contemplating this program.

